# Storytelling and poetry in the time of coronavirus: medical students’ perspective

**DOI:** 10.1017/ipm.2020.120

**Published:** 2020-11-04

**Authors:** Edward Antram, Ella Burchill

**Affiliations:** Faculty of Medicine and Life Sciences, King’s College London, London, UK

Dear Editor,

We were interested to read your recently published paper describing the need for storytelling and poetry for clinicians in the time of coronavirus (Barrett *et al.* 2020). As medical students, this necessity for storytelling strongly resonated with us: helping us become emotionally literate clinicians whilst enabling a better understanding of patients’ experience.

In the original paper, it was highlighted that clinicians and patients can use poetry and storytelling to ‘support identification’, ‘to process experiences’ and ‘to promote coping’. This is especially important during the intense and emotional pandemic period, when this narrative approach to practicing medicine can aid in building resilience for clinicians. However, we feel when applied to the context of medical student education, these qualitative mediums enable us to develop self-knowledge and to deepen the insight required as clinicians to have effective and compassionate patient communication.

For medical students, the COVID-19 pandemic has introduced new challenges for existing curricula and in gaining adequate clinical experiences. In a learning environment now dominated by virtual lectures, self-directed learning and a lack of patient contact, a narrative approach for learning medicine is more important than ever. We believe integrating narratives robustly into the curriculum is a priority; fostering these important skills early will help our future doctors become more reflective, conscientious and resilient in their practice.

All the world’s a stage,

And all the men and women are merely players;

– Shakespeare, As You Like It, Act II Scene 7

Narrative is fundamental to understanding human experience. In medicine, ‘players’ are regularly put in situations that challenge emotions and conceptions of self; often requiring difficult decisions. Clinicians’ actions should balance the rational mechanisms of procedural medicine and the requirement to understand the meaning of our roles and interactions. Thus, as clinicians, it is fundamental to develop narrative competence (Charon 2001). Literature and poetry allow us to ‘sharpen listening, attentiveness, observation and analytical skills’, whilst also enhancing ‘empathy, self-awareness and introspection’ (Bromberg 2008). Literature and poetry are, therefore, an ideal instrument to develop one’s narrative competence.

Barrett *et al*., explain that narrative competence can be sought and practiced using several approaches, including poetry, storytelling and literature in addition to Schwartz Rounds and Balint groups. We will analyse and discuss these techniques for medical students – for whom the utility and benefits likely differ from those of a clinician.

Literature and poetry are a valuable tool in developing medical students into self-realised, emotionally conscious clinicians. Existing programmes have improved communication skills and clinician-patient relationships (Brown 2015). Used in combination with narrative learning in modern medical curricula, such as Balint groups and Schwartz Rounds – this offers an opportunity for students to develop skills that will serve both patients and their future emotional wellbeing. Implementing medical humanities into the curriculum allows reflections upon the transformations that inevitably arise with the job, and the multifaceted professionalism these require (Shafer *et al.* 2007). The value of literature within medical education is its focus on empathy from cognitive and affective perspectives, beyond just the active component of feeling listened to and understood (Wolters and Wijnen-Meijer 2012). Notable examples of medical students’ poetry have been produced by Bristol students (Gardner *et al.* 2010; Thompson *et al.* 2010). An appreciation of poetry in medicine exists throughout the profession with poetry published in scientific journals and the success of the Hippocrates Prize for Poetry and Medicine. With such pivotal roles for sustaining empathy and communication skills, poetry and literature are a powerful adjunct to reflective and clinical practice and medical curricula should prioritise them as such.

As undergraduates, we were fortunate to experience Balint groups. These were a valuable forum to discuss and reflect upon emotionally challenging clinical situations, develop interpersonal skills and to view the patient beyond a clinical problem-solving exercise. Patient narratives at medical school can sadly exist merely as simplified ‘cases’ and patient presentations are only discussed at surface level. To develop meaningful doctor–patient relationships, exploring patient narratives should be prioritised alongside learning communication and history taking skills. Recent research has highlighted the value of Balint groups for undergraduates, improving personal and professional development, stress management and reducing burnout (Yazdankhahfard *et al*. 2019). Awareness of the doctor–patient relationship, communication skills and reflecting on our own prejudices and psychological factors can also be effectively explored in this setting (Yakeley *et al.* 2011; O’Neill *et al*. 2016).

Schwartz Rounds foster a compassionate culture; through which clinicians feel patient-centred care is prioritised (Goodrich 2012; Francis 2013). Incorporating rotating students into Schwartz Rounds within a longstanding clinical team may challenge the safe environment created for open discussion. Nonetheless, implementing Schwartz Rounds into the undergraduate curriculum has been well-received by medical students, promoting empathy, resilience and communication skills (Shield *et al.* 2011; Barker *et al*. 2016; Gishen *et al.* 2016; Stocker *et al.* 2018). This approach is especially pertinent when a loss of empathy has been reported by medical students as they progress through clinical phases of training (Hojat *et al.* 2020).

Balint groups and Schwartz Rounds can therefore be seen as effective for students to develop self-awareness, in addition to promoting personal growth and well-being. Whilst these techniques promote self-exploration in a safe setting, literature and poetry allow the first-hand experience of multiple and diverse voices, fostering creativity and acceptance of ambiguity. Medical schools should promote these values to prepare our future doctors to face the uncertainties and pressures of the clinical workplace (Novack *et al*. 1999; Schön 2017).

Barrett *et al*., express the need for ‘new frameworks of systemic support’ in response to increasing clinician burnout. In response, we would like to highlight increasing burnout amongst medical students: a recent study found 29% of respondents were given a mental health diagnosis during medical school, and 85% could be classified as ‘disengaged’ and 85% ‘exhausted’ (Farrell *et al.* 2019). Many medical students are at risk of or already suffering from burnout before qualifying (Cecil *et al.* 2014). As students progress through training, beyond the risk to clinician’s own personal health, this also has implications for patient care, communication and outcomes (Dyrbye *et al.* 2006; Halbesleben and Rathert 2008; Brown *et al.* 2009). Preventative intervention is key to stop burnout before its initiation.

Thus, establishing narrative learning is paramount for medical students, this should be done not only through Balint groups and Schwartz Rounds, but more importantly through the cultivation of a culture of self-knowledge and lifelong learning. It is our view, motivated by Barrett *et al*., that learning narrative techniques at medical school will help students develop not only into effective, compassionate clinicians, but also into self-realised human beings.

## References

[ref1] Barker R , Cornwell J , Gishen F (2016). Introducing compassion into the education of health care professionals: can Schwartz Rounds help? Journal of Compassionate Health Care 3, 3. doi: 10.1186/s40639-016-0020-0

[ref2] Barrett E , Dickson M , Hayes-Brady C , Wheelock H (2020). Storytelling and poetry in the time of coronavirus. Irish Journal of Psychological Medicine. doi: 10.1017/ipm.2020.36. PMC7387760 32406356

[ref3] Bromberg R (2008). Poetry and medicine. Medscape General Medicine 10, 63. PMC2329765 18449350

[ref4] Brown R , Dunn S , Byrnes K , Morris R , Heinrich P , Shaw J (2009). Doctors’ stress responses and poor communication performance in simulated bad-news consultations. Academic Medicine 84, 1595–1602. doi: 10.1097/ACM.0b013e3181baf537. 19858823

[ref5] Brown SA (2015). Creative expression of science through poetry and other media can enrich medical and science education. Frontiers in Neurology 6, 3. doi: 10.3389/fneur.2015.00003. 25657637 PMC4302945

[ref6] Cecil J , McHale C , Hart J , Laidlaw A (2014). Behaviour and burnout in medical students. Medical Education Online 19. doi: 10.3402/meo.v19.25209. PMC4145104 25160716

[ref7] Charon R (2001). Narrative medicine: a model for empathy, reflection, profession, and trust. Journal of the American Medical Association 286, 1897–1902. doi: 10.1001/jama.286.15.1897. 11597295

[ref8] Dyrbye LN , Thomas MR , Huntington JL , Lawson KL , Novotny PJ , Sloan JA , Shanafelt TD (2006). Personal life events and medical student burnout: a multicenter study. Academic Medicine 81, 374–384. doi: 10.1097/00001888-200604000-00010. 16565189

[ref9] Farrell SM , Kadhum M , Lewis T , Singh G , Penzenstadler L , Molodynski A (2019). Wellbeing and burnout amongst medical students in England. International Review of Psychiatry 31, 579–583. doi: 10.1080/09540261.2019.1675960. 31692396

[ref10] Francis R (2013). Report of the Mid Staffordshire NHS Foundation Trust Public Inquiry, New Directions for Youth Development. London: The Stationery Office.

[ref11] Gardner C , Golestaneh L , Dhillon B , Shafer A (2010). People say there are no accidents: poetry and commentary. Journal of Medical Humanities 31, 257–263. doi: 10.1007/s10912-010-9113-5. 20414798

[ref12] Gishen F , Whitman S , Gill D , Barker R , Walker S (2016). Schwartz Centre Rounds: a new initiative in the undergraduate curriculum – what do medical students think? BMC Medical Education 16, 246. doi: 10.1186/s12909-016-0762-6. 27658411 PMC5034622

[ref13] Goodrich J (2012). Supporting hospital staff to provide compassionate care: do Schwartz Center Rounds work in English hospitals? Journal of the Royal Society of Medicine 105. doi: 10.1258/jrsm.2011.110183. PMC3308638 22434811

[ref14] Halbesleben JRB , Rathert C (2008). Linking physician burnout and patient outcomes: exploring the dyadic relationship between physicians and patients. Health Care Management Review 33, 29–39. doi: 10.1097/01.HMR.0000304493.87898.72. 18091442

[ref15] Hojat M , Shannon SC , DeSantis J , Speicher MR , Bragan L , Calabrese LH (2020). Does empathy decline in the clinical phase of medical education? A nationwide, multi-institutional, cross-sectional study of students at DO-granting medical schools. Academic Medicine 95, 911–918. doi: 10.1097/ACM.0000000000003175. 31977341 PMC7242173

[ref16] Novack DH , Epstein RM , Paulsen RH (1999). Toward creating physician-healers: fostering medical students’ self- awareness, personal growth, and well-being. Academic Medicine, 516–520. doi: 10.1097/00001888-199905000-00017. 10353283

[ref17] O’Neill S , Foster K , Gilbert-Obrart A (2016). The Balint group experience for medical students: a pilot project. Psychoanalytic Psychotherapy 30, 96–108. doi: 10.1080/02668734.2015.1107124.

[ref18] Schön DA (2017). The Reflective Practitioner: How Professionals Think in Action. London: Routledge. doi: 10.4324/9781315237473.

[ref19] Shafer A , Maxwell B , Strauss R , Kopp VJ (2007). I must tell you in a poem: poetry and commentary. Journal of Medical Humanities 28, 173–180. doi: 10.1007/s10912-007-9037-x. 17554611

[ref20] Shield RR , Tong I , Tomas M , Besdine RW (2011). Teaching communication and compassionate care skills: an innovative curriculum for pre-clerkship medical students. Medical Teacher 33, e408–e416. doi: 10.3109/0142159X.2011.586748. 21774636

[ref21] Stocker C , Cooney A , Thomas P , Kumaravel B , Langlands K , Hearn J (2018). Schwartz rounds in undergraduate medical education facilitates active reflection and individual identification of learning need. MedEdPublish. doi: 10.15694/mep.2018.0000230.1. PMC10712006 38089201

[ref22] Thompson T , van de Klee D , Lamont-Robinson C , Duffin W (2010). Out of our heads! Four perspectives on the curation of an on-line exhibition of medically themed artwork by UK medical undergraduates. Medical Education Online 15. doi: 10.3402/meo.v15i0.5395. PMC3037266 21321667

[ref23] Wolters FJ , Wijnen-Meijer M (2012). The role of poetry and prose in medical education: the pen as mighty as the scalpel? Perspectives on Medical Education 1, 43–50. doi: 10.1007/s40037-012-0008-1. 23316458 PMC3540367

[ref24] Yakeley J , Shoenberg P , Morris R , Sturgeon D (2011). Psychodynamic approaches to teaching medical students about the doctor-patient relationship: randomised controlled trial. Psychiatrist 35, 308–313. doi: 10.1192/pb.bp.110.033704.

[ref25] Yazdankhahfard M , Haghani F , Omid A (2019). The Balint group and its application in medical education: a systematic review. Journal of Education and Health Promotion 8, 124. doi: 10.4103/jehp.jehp_423_18. 31334276 PMC6615135

